# Priority areas for conservation alone are not a good proxy for predicting the impact of renewable energy expansion

**DOI:** 10.1073/pnas.2204505119

**Published:** 2022-07-25

**Authors:** Juan M. Pérez-García, Jon Morant, Eneko Arrondo, Esther Sebastián-González, Sergio A. Lambertucci, Andrea Santangeli, Antoni Margalida, José A. Sánchez-Zapata, Guillermo Blanco, José A. Donázar, Martina Carrete, David Serrano

**Affiliations:** ^a^Department of Applied Biology, Miguel Hernández University, 03202 Elche, Spain;; ^b^Department of Ornithology, Aranzadi Sciences Society, 20014 Donostia-San Sebastián, Spain;; ^c^Department of Ecology, University of Alicante, 03690 Alicante, Spain;; ^d^Conservation Biology Research Group, Instituto de Investigaciones en Biodiversidad y Medioambiente (Consejo Nacional de Investigaciones Científicas y Técnicas (INIBIOMA-CONICET), Universidad Nacional del Comahue, 1250 Bariloche, Argentina;; ^e^Research Centre for Ecological Change, Organismal and Evolutionary Biology Research Programme, University of Helsinki, 00100 Helsinki, Finland;; ^f^FitzPatrick Institute of African Ornithology, Department of Science and Technology-National Research Foundation Centre of Excellence (DST-NRF), University of Cape Town, 7701 Cape Town, South Africa;; ^g^Institute for Game and Wildlife Researchc (IREC), Consejo Superior de Investigaciones Científicas-Universidad de Castilla-La Mancha (CSIC-UCLM), 13005 Ciudad Real, Spain;; ^h^Department of Biodiversity Conservation, Pyrenean Institute of Ecology, Consejo Superior de Investigaciones Científicas (CSIC), 22700 Jaca, Spain;; ^i^Department of Evolutionary Ecology, National Museum of Natural Sciences, Consejo Superior de Investigaciones Científicas (CSIC), 28006 Madrid, Spain;; ^j^Department of Conservation Biology, Estación Biológica de DoñanaConsejo Superior de Investigaciones Científicas (CSIC), 41092 Sevilla, Spain;; ^k^Department of Physical, Chemical and Natural Systems, Universidad Pablo de Olavide, 41013 Sevilla, Spain

There is broad consensus that increasing the use of renewable energies is effective to mitigate the global climate crisis. However, the development of renewables may carry environmental impacts, and their expansion could accelerate biodiversity loss (1). However, Dunnett et al. (2) have recently estimated a minimal overlap between renewable energy expansion and important conservation areas (ICAs; i.e., protected areas, key biodiversity areas, wilderness areas) (sensu ref. 2), suggesting that these infrastructures would not significantly affect biodiversity conservation if properly planned and regulated.

**Fig. 1.**
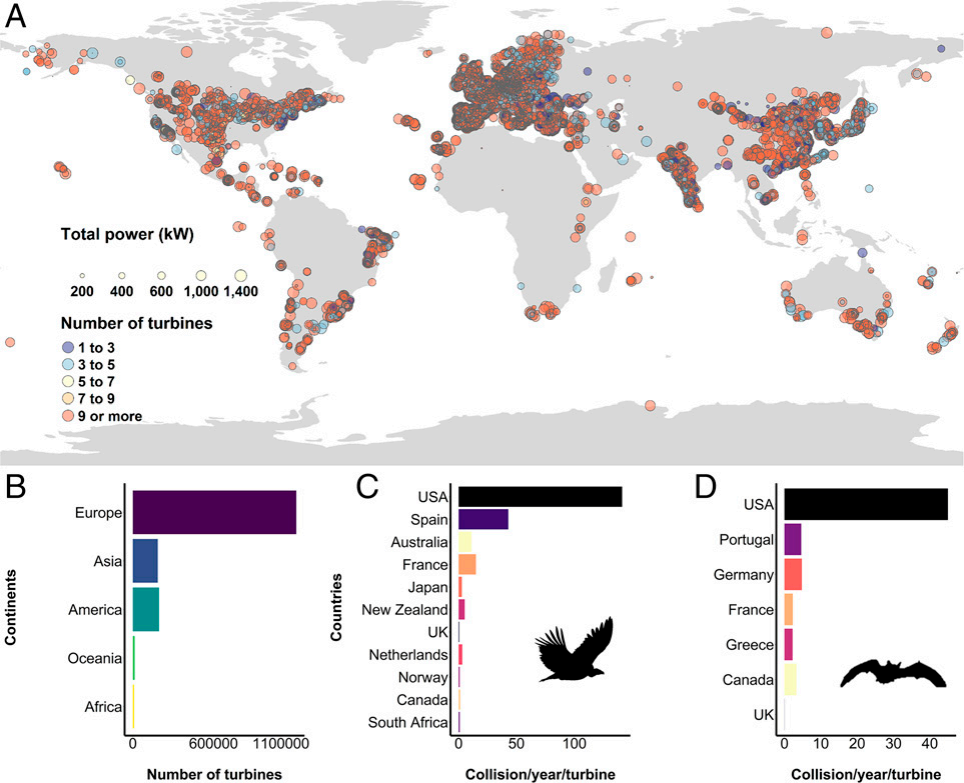
Worldwide distribution of wind farms in which the total power generated (kilowatts) and the number of turbines are represented by dot size and color, respectively (*A*). *B–D* represent the total number of turbines per continent (*B*) and collisions per year per turbine in each country for birds (*C*) and bats (*D*) based on the data from Thaxter et al. (3).

Assessing the impacts of renewables on biodiversity only in terms of their spatial overlap with ICAs ignores that these impacts on species and functional groups are asymmetric. Long-lived species are highly vulnerable to the loss of specific habitats or to nonnatural mortality, and these factors should be considered when studying conflicts between renewables and biodiversity (3). For instance, one of the most concerning impacts of wind farms, which have dramatically multiplied worldwide in recent years (Fig. 1 *A* and *B*), is the nonnatural mortality of highly mobile flying species, such as birds (4) and bats (5), due to collisions with turbines (Fig. 1 *C* and *D*). Many of these species spend a large part of their life cycle outside ICAs (6, 7), where mortality caused by infrastructures can extirpate populations at regional scales and even within ICAs (8). Consequently, thinking that we can rely only on ICAs for the protection of these species is very risky and may obscure the real magnitude of the threat posed by renewable energy development (7).

**Fig. 2.**
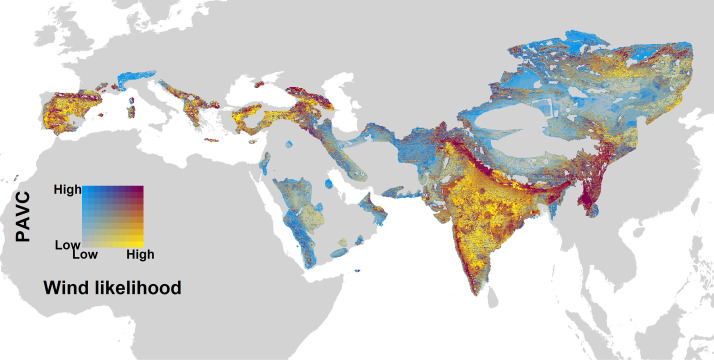
Overlap between priority areas for Old World vulture conservation (PAVC) and wind expansion likelihood. Magenta cells represent the highest risk of impacts with wind farms. Wind likelihood is the predicted probability (zero to one) that an energy installation is present in a given grid cell (taken from ref. 2). PAVC ranks global cells from low to high priority (zero to one) according to the breeding and resident range of the 15 Eurasian vulture species (taken from ref. 8).

Vultures, 70% of which are threatened (9), provide a clear example of how wind energy development outside ICAs continues to pose a threat. Analyzing the overlap between priority areas for Eurasian vulture conservation (8) and wind speed (which is an indicator of wind energy expansion) (2), we found that 31% of these vulture conservation areas (9) overlap by more than 80% with the expected expansion of wind energy (Fig. 2). Based on extensive available research (3, 4, 7, 10), we can anticipate that the expansion of wind energy outside ICAs will represent a high threat to vultures and other large soaring birds. Similar threats are likely applied to other less studied but also highly sensitive species, such as bats, which spend much of their lifetime outside ICAs (5). Regarding solar energy facilities, they occupy agricultural areas located mostly outside ICAs (1), and their expansion would seriously compromise the persistence of most European steppe birds, which occupy agricultural areas located mostly outside ICAs (1).

Thus, while we agree with Dunnett et al. (2) on the need for a transition to decarbonization of energy through, among other things, the promotion of renewables, we believe that the potential impacts of these infrastructures need to be predicted more comprehensively. In the meantime, claiming that the “minimal overlap” between renewables and ICAs means that their impacts on biodiversity will be low carries a high risk of fueling greenwashing of otherwise sustainable energy sources.
